# Supplementary material for: Neuron-Radial Glial Cell Communication via BMP/Id1 Signaling Is Key to Long-Term Maintenance of the Regenerative Capacity of the Adult Zebrafish Telencephalon
Source: Cells. 2021 Oct 19;10(10):2794. doi: 10.3390/cells10102794 (PMC8534405; doi:10.3390/cells10102794)
Supplement: Supplementary file 1 [file cells-10-02794-s001.zip › cells-1409959-supplementary.pdf]

Supplementary figures and legends

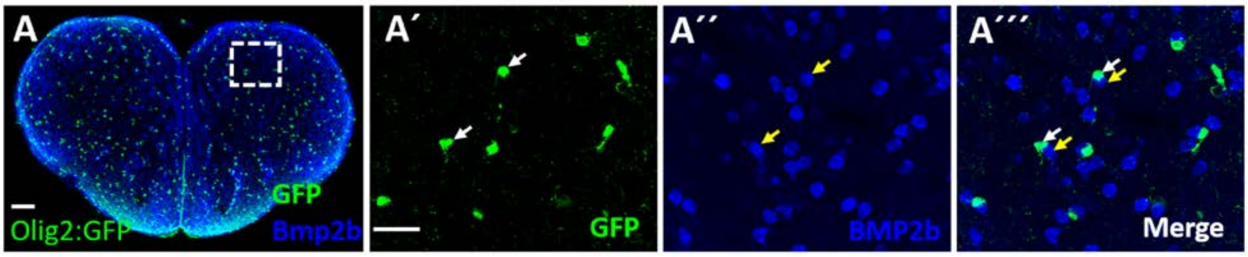

Figure S1. BMP2b is not detectably expressed in oligodendrocytes of the telencephalon. Cross-sections through the telencephalon of *Tg(olig2:gfp)* transgenic fish marking oligodendrocytes (green) were immunostained with a BMP2b antibody (blue). The boxed-in area in A is magnified in A’ to A’’’ showing individual channels (A’, A’’) and the merged image (A’’’). White arrows show 2 oligodendrocytes, yellow arrows show BMP2b+ neurons. Scale bars: 100 µm (A), 20 µm (A’-A’’’).

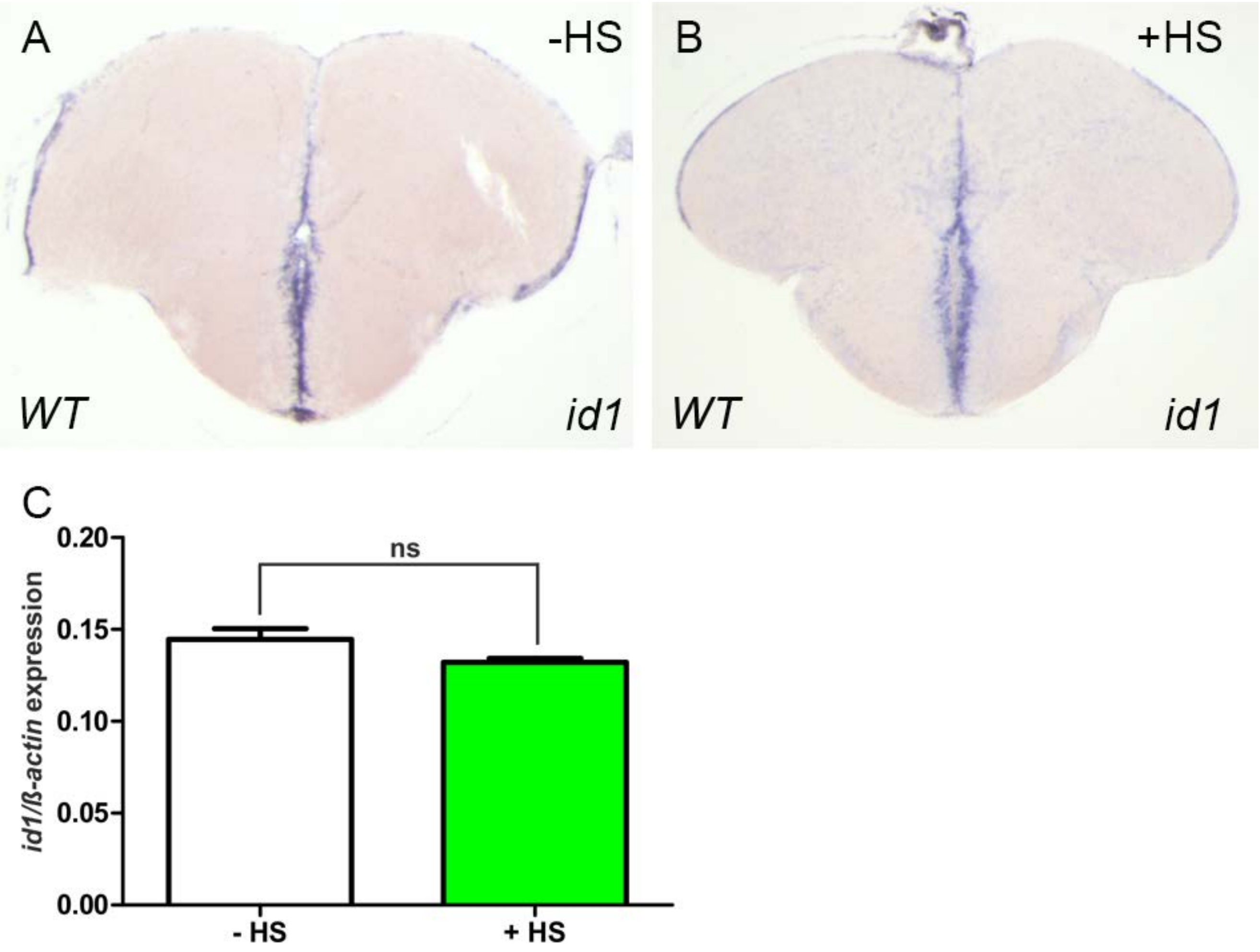

Figure S2. Heat-shock has no influence on the endogenous expression of the *id1* gene.

(A-B) ISH against *id1* on cross- sections of WT telencephala. There is no difference in the expression level for *id1* between non heat-shocked (A) and heat-shocked (B) WT brains. (C) RT-qPCR analysis of *id1* mRNA expression levels in WT with and without heat-shock reveals that the level of *id1* mRNA does not change in response to heat-shock in the WT brain. n=3 brains. Significance is indicated by ns, not significant.

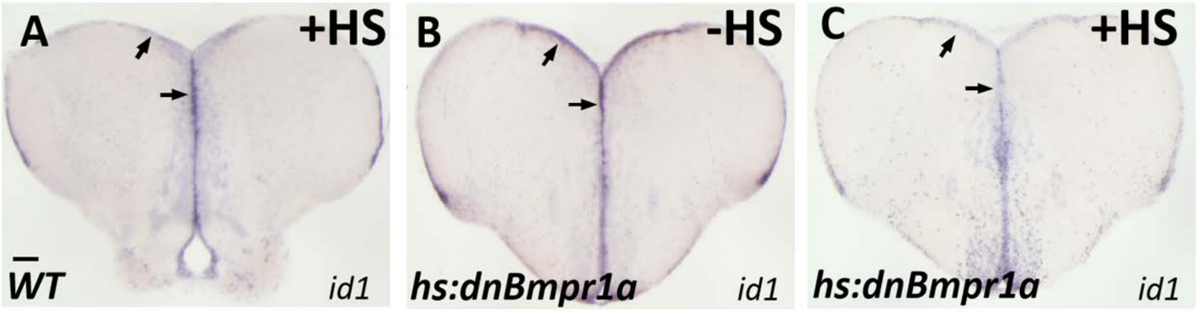

Figure S3. Heat-shock does not influence the expression pattern of *id1* in WT fish.

(A-C) Cross-sections through telencephala of heat-shocked WT, (A) and *hs:dnBMPR1a* transgenic animals without (B) and with heat-shock (C). Heat-shock of WT animals did not influence the expression of *id1*. In contrast, decrease of *id1* expression was noted after inhibition of the BMP pathway by heat-shock of *Tg(hs: dnBmpr1a)* animals (C) relative to transgenics without heat-shock (B). Black arrows show the expression of *id1* in the ventricular zone of WT (A) and *Tg(hs:dnBmpr1a)* animals without heat-shock (B) and after heat-shock (C). n=3 animals (A-C). Scale bar: 20 µm. HS, heat-shock.

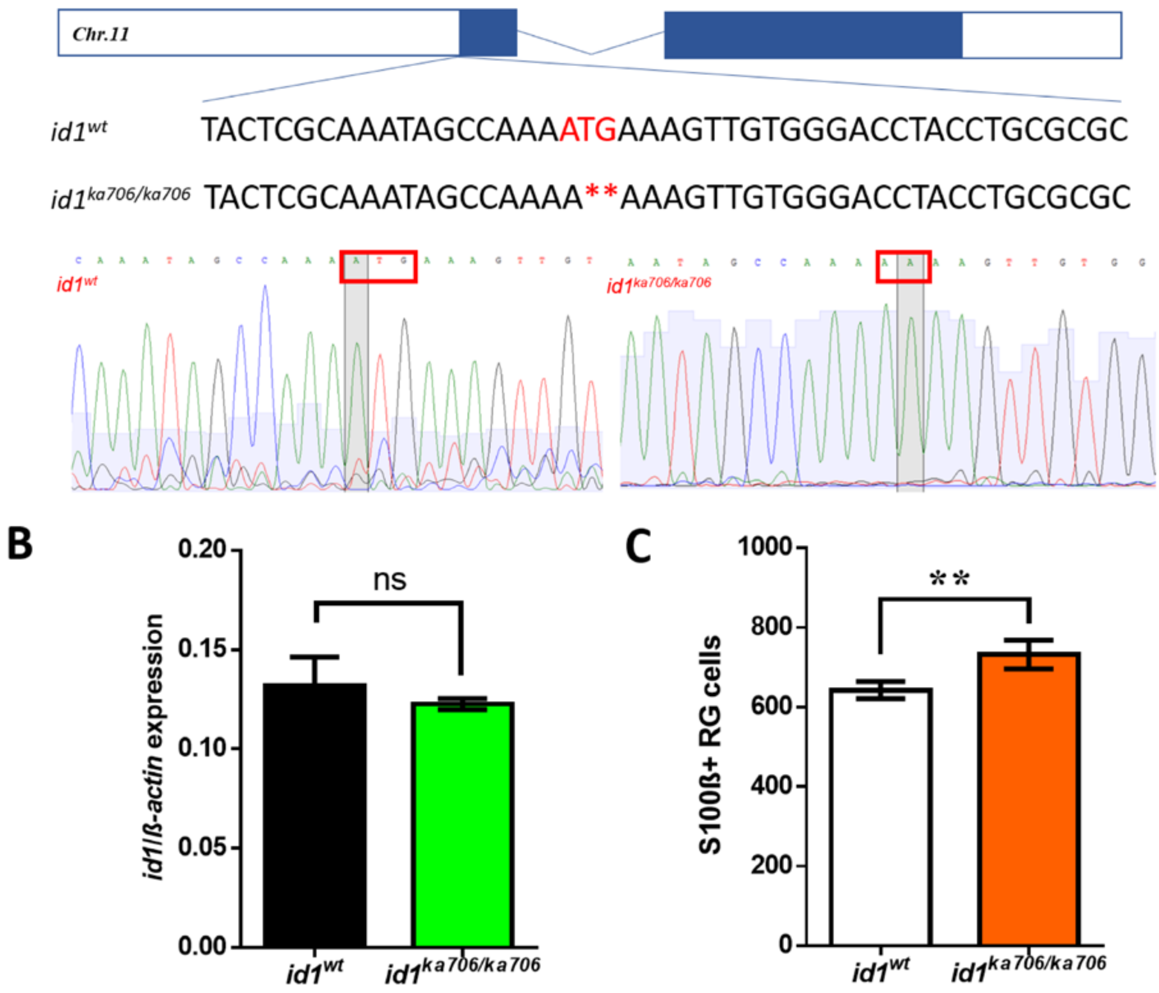

Figure S4. Generation of a CRISPR/Cas 9-mediated Id1 loss- of- function mutant *id1*$^{ka706/ka706}$. (A) Schematic representation of the *id1* locus on chromosome 11. The gene consists of 2 exons. Red letters represent the start codon of the *id1* coding sequence. The Sanger sequencing results of WT (*id1*^{WT}) and homozygous (*id1*$^{ka706/ka706}$) sequences are displayed underneath. In the homozygous sequences T and G of the start codon are deleted. (B) RT-qPCR analysis of *id1* mRNA expression levels in WT and *id1*$^{ka706/ka706}$ telencephala reveals that the level of *id1* mRNA is not changed in the mutant. (C) Quantification of the S100β+ RGCs/NSCs in WT and *id1*$^{ka706/ka706}$ telencephala without injury. n=3 brains (B, C). Significance is indicated by asterisks: ns, not significant; **, p <0.01.

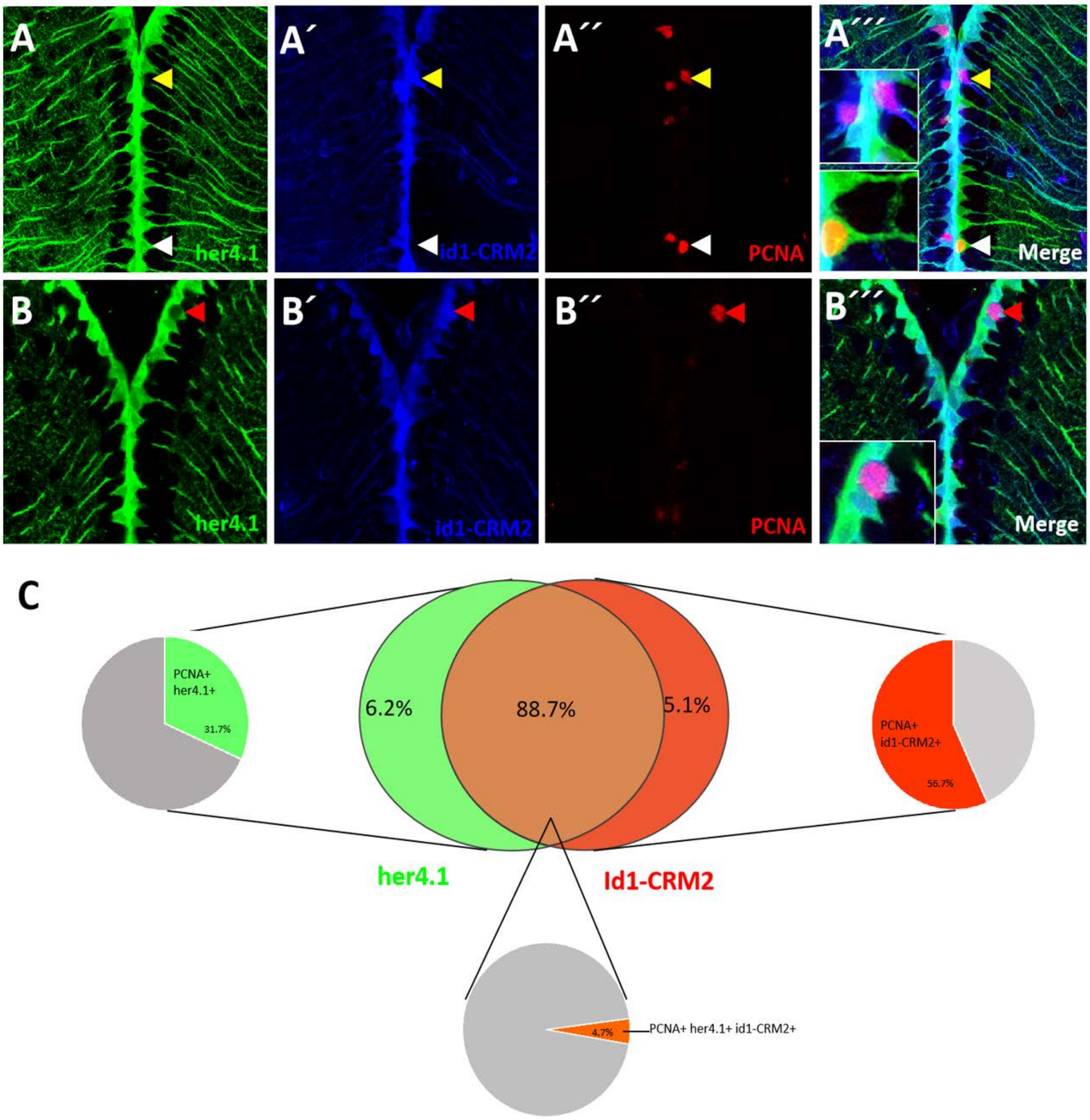

Figure S5. Both *notch3* and *her4.1* are expressed together with *id1* in the RGCs of the telencephalon. (A-B’’’) Immunostaining on cross sections of the telencephalon of *Tg(her4.1:gfp;id1-CRM2:mCherry)* double transgenics with antibodies against GFP (green), mCherry (blue) and the proliferation marker PCNA (red). GFP and mCherry signals co-localize indicating that *her4.1* and *id1* are co-expressed. A’’’ inset: magnified image of PCNA+/id1-CRM2:mCherry+ cells (yellow arrowhead, upper image) and a PCNA+/her4.1+ cell (white arrowhead, lower image). B’’’ inset: a magnified view of a PCNA+/id1-CRM2:mCherry+/her4.1:gfp+ cell (red arrowhead). (C) Summary of co-expression analysis of (*Tg(id1-CRM2:mCherry)*, red) and (*Tg(her4.1:gfp*), green). Pie charts represent total number

of *Tg(her4.1:gfp)* or *Tg(id1-CRM2:mCherry)* or both *Tg(her4.1:gfp)* and *Tg(id1-CRM2:mCherry)* expressing cells and the fraction of cells co-expressing PCNA. Cells expressing either *Tg(id1-CRM2:mCherry)* alone or *Tg(her4,1:gfp)* alone show a higher proportion of PCNA+ cells than cells expressing both markers. Scale bar = 20 µm. n=3 brains.

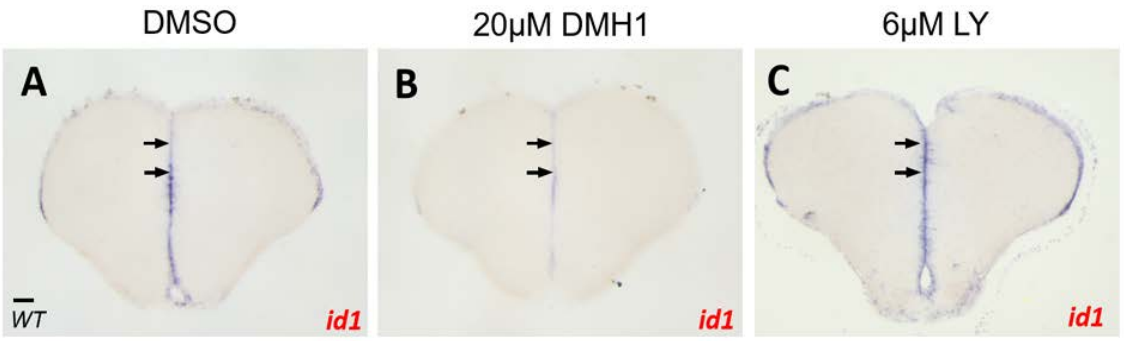

Figure S6. Expression of *id1* is independent of Notch signaling

(A-C) Expression of *id1* revealed by ISH on control brains (DMSO, A) or brains treated with different concentrations of DMH1 (B) or LY (C). (B) Inhibition of BMP signaling by 20 µM DMH1 lead to the reduction of *id1* expression. (C) Inhibition of Notch signaling by 6 µM LY411575 did not influence the expression of *id1* but strongly reduced *her4.1* expression (See Fig, 7C). Black arrows show the expression of *id1* in the ventricular zone of untreated (A) and treated (B and C) adult zebrafish telencephala. n=3 brains. Scale bar: 100 µm.

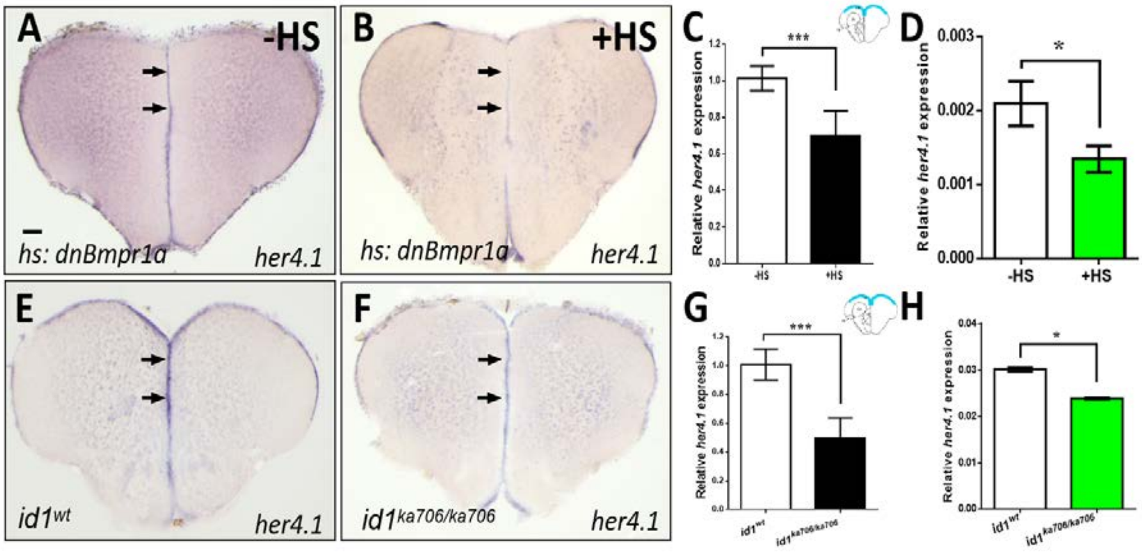

Figure S7. Inhibition of BMP signaling or deletion of *id1* leads to a reduction of *her4.1* expression.

(A-B) ISH reveals decrease of *her4.1* expression after inhibition of the BMP pathway by heat-shock of *Tg(hs:dnBmpr1a)* animals. Black arrows show the expression of the *her4.1* gene in the ventricular zone of *Tg(hs:dnBmpr1a)* animals without (A) and with heat-shock (B). (C) Quantification of *her4.1* expression in panels A and B (scheme in the upper right-hand corner displays the quantified area in blue). (D) RT-qPCR quantification of *her4.1* mRNA expression of brains shown in A-B. (E-F) Expression of *her4.1* mRNA is reduced in $id1^{ka706/ka706}$ telencephala. Black arrows show the region of expression of *her4.1* in the ventricular zone of $id1^{ka706/ka706}$ mutants and WT siblings. (G) Quantification of *her4.1* expression (scheme in the upper right-hand corner displays the quantified area in blue). (H) RT-qPCR quantification confirms reduction of *her4.1* in $id1^{ka706/ka706}$ telencephala. HS, heat-shock. n=3 independent brains (C, G), n=15 sections D, H

Table S1. Genes co-expressed with *id1+/ccdn1-* RGCs/NSCs according to single cell sequencing data from (41)**.**

Name	ID	Pearson_cor	Function	Reference
smad1	ENSDARG00000027199	0.448256850	signal transducer	(81)
acvr2b	ENSDARG00000044422	0.379378967	activin receptor type II	(82)
smad5	ENSDARG00000037238	0.323494119	signal transducer	(81)
bambia	ENSDARG00000055381	0.222851826	target gene and modulator	(83)
bmpr2b	ENSDARG00000020057	0.191787248	Bmp type II receptor	(73)
acvr2aa	ENSDARG00000011188	0.181544807	activin receptor type II	(82)
bmpr1ab	ENSDARG00000105045	0.088841975	Bmp type I receptor	(75)
smad9	ENSDARG00000021938	0.061794402	signal transducer	(39)

Table S2. Number of WT and *id1*$^{ka706/ka706}$ adult zebrafish used in the multiple stab wound experiments.

	Start number	Fish sacrificed at 5dpl	Fish sacrificed after second stab injury	Fish survived after 3 months
id1^{wt}	25	2	7	16
id1$^{ka706/ka706}$	25	2	7	11

Table S3. PCR primer sequences for ISH probes

bmp2a Forward	5'-ACGCAGAGCAGGTTAGCA-3'
bmp2a Reverse	5'- GAAGGGACCGACTTACGC-3'
bmp7a Forward	5'-CAGTTGCTGCTGATTTGTTG-3'
bmp7a Reverse	5'-TGAACATCTGTGAGGGGATT-3'
bmp7b Forward	5'-CAGGTTGTGCGCTCTGGAAACGCAA-3'
bmp7b Reverse	5'-CCTTTTGCAATCCTGGCCAATCG-3'
bmpr2b Forward	5'- TATTGTCGCGCTGTTCTTTG-3'
bmpr2b Reverse	5'- GCAGATAGGCCAGTCCTCTG-3'

Table S4. Restriction enzymes and RNA polymerases used for DIG probe synthesis:

Gene name	Restriction enzyme	RNA-polymerase
id1	XhoI	T3
her4.1	CeuI	T7
notch3	ApaI	SP6
bmp2a	ApaI	SP6
bmp2b	SpeI	T7
bmp4	BamHI/SpeI	T7
bmp7a	SacII	SP6
bmp7b	NcoI	SP6
bmpr1ab	EcoRi	T7
bmpr2b	PstI	T7

Table S5. PCR primer sequences for RT-qPCR.

ß-actin RT Fwd	5´-GCC TGA CGG ACA GGT CAT-3´
ß-actin RT Rev	5´-ACC GCA AGA TTC CAT ACCC-3´
ascl1a RT Fwd	5´-ATC TCC CAA AAC TAC TCT AAT GAC ATG AAC TCT AT-3´
ascl1a RT Rev	5´-CAA GCG AGT GCT GAT ATT TTT AAG TTT CCT TTT AC-3´
her4.1 RT Fwd	5´-GCT GAT ATC CTG GAG ATG ACG-3´
her4.1 RT Rev	5´-GAC TGT GGG CTG GAG TGT GTT-3´
id1 RT *Fwd*	5´-ATG TTG TCC GCT GCC TCT-3´
id1 RT *Rev*	5´-GCT GGC TTT CTT GTT GGT C-3´

References

39. Wei CY, Wang HP, Zhu ZY, Sun YH. Transcriptional factors smad1 and smad9 act redundantly to mediate zebrafish ventral specification downstream of smad5. J Biol Chem. 2014;289(10):6604-18.
73. Monteiro R, van Dinther M, Bakkers J, Wilkinson R, Patient R, ten Dijke P, et al. Two novel type II receptors mediate BMP signalling and are required to establish left-right asymmetry in zebrafish. Dev Biol. 2008;315(1):55-71.
75. Smith KA, Noel E, Thurlings I, Rehmann H, Chocron S, Bakkers J. Bmp and nodal independently regulate lefty1 expression to maintain unilateral nodal activity during left-right axis specification in zebrafish. PLoS Genet. 2011;7(9):e1002289.
81. Muller F, Blader P, Rastegar S, Fischer N, Knochel W, Strahle U. Characterization of zebrafish smad1, smad2 and smad5: the amino-terminus of smad1 and smad5 is required for specific function in the embryo. Mech Dev. 1999;88(1):73-88.
82. Albertson RC, Payne-Ferreira TL, Postlethwait J, Yelick PC. Zebrafish acvr2a and acvr2b exhibit distinct roles in craniofacial development. Dev Dyn. 2005;233(4):1405-18.
83. Tsang M, Kim R, de Caestecker MP, Kudoh T, Roberts AB, Dawid IB. Zebrafish nma is involved in TGFbeta family signaling. Genesis. 2000;28(2):47-57.
